# Complete mitochondrial genome sequence of *E. suratensis* revealed by next generation sequencing

**DOI:** 10.1080/23802359.2016.1176877

**Published:** 2016-11-21

**Authors:** Sudip K. Mohanta, Subrat K. Swain, Sofia P. Das, Amrita Bit, Gargee Das, Sanghamitra Pradhan, Jitendra K. Sundaray, P. Jayasankar, A. S. Ninawe, Paramananda Das

**Affiliations:** a Department of Zoology, Banabhumi (Degree) Mahavidyalaya, Rangamatia, Odisha, India;; b Fish Genetics and Biotechnology Division, ICAR-Central Institute of Freshwater Aquaculture, Bhubaneswar, Odisha, India;; c Department of Biotechnology, Goverment of India, New Delhi, India

**Keywords:** *Etroplus suratensis*, mitogenome, cichlidae, NGS method, sequence

## Abstract

The complete mitochondrial genome of *Etroplus suratensis*, the Green chromide cichlid, was determined for the first time through NGS method. The genome is 16,467 bp (Accession no. KU301747) in length and consisted of 13 protein-coding genes, 22 tRNA genes, 2 rRNA genes and one control region. Organization of genes and their order are in accordance with other vertebrates. The overall base composition on plus strand was A: 28.3%, G: 15.2%, C: 30.9%, T: 25.6%, and the A + T content 53.9%. The control region contains a putative termination-associated sequence and three conserved sequence blocks. This mitogenome sequence data would play an important role in population genetics and phylogenetics of cichlid fish of India.

*Etroplus suratensis* (Bloch 1790), belonging to the family cichlidae, is a medium-sized cichlid fish commonly distributed in the brackish water lakes, reservoirs and back waters in India and Srilanka. In India it is inhabitant of brackish water lakes, reservoirs of Kerala, Karnataka, Andhra Pradesh and Chilika lake of Odisha. Wild population of *Etroplus suratensis* is under pressure due to habitat deterioration, introduction of exotic species (Kurup & Radhakrishnan 2006; Krishnakumar et al. 2009) and outbreak of Epizootic Ulcerative Syndrome (EUS) in South & South-east Asia (Pathiratne & Rajapakshe 1998). The wild population of this species has not given sufficient conservation attention and it belongs to the IUCN red list of threatened species (Abraham 2013). Therefore, development of genomic resources including complete michondrial genome is the need of hour. The aim of the present study was to get insight into the mitochondrial genome structure, evolution and phylogeny of cichlid fish. So the complete mitochondrial genome sequence of *E. suratensis* was deduced. It is expected that the information obtained from complete mitochondrial genome sequence of *E. suratensis* would provide a useful genetic resources to be utilized in the future investigation on population genetics and phylogenomics of cichlid fish. DNA from 100mg fin tissue of a single specimen of *E. suratensis* weighing approximately 250g collected from Chilika lake (19.8450°N, 85.4788°E) was extracted by standard phenol-chloroform extraction method (Sambrook & Russell 2001) and rest of the sample was preserved in −80 °C at ICAR-Central Institute of Freshwater Aquaculture with the voucher no. ES001. DNA sequencing was prepared in paired-end libraries, tagged and subjected to next generation sequencing (NGS) on the Illumina Next-Seq 500 system (Genotypic Technology Pvt. Ltd, Bengaluru, India). Quality check, raw read pre-processing and *de novo* assembly was performed using CLC Genomics Workbench version 7.0.4 (Aarhus, Denmark). Characterization of *E. suratensis* mitogenome was carried out by comparing with a closely related fish mitogenome (Saitoh et al. 2011). A phylogenetic tree was constructed based on 10 representatives from Cichlidae family and one cyprinidae, complete mtDNA sequence (Figure 1).

**Figure 1.**
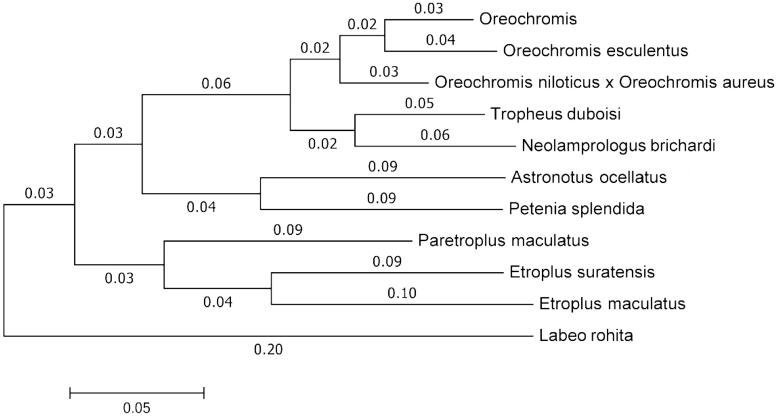
Molecular phylogeny of *E. suratensis* and other related species in Cichlidae based on the complete mitogenome.

The complete mitogenome of *E. suratensis* is 16,467 bp in length. It consists of 13 protein-coding genes, 22 tRNAs, two rRNA genes and one putative control region and the sequence has been submitted to the GenBank (Accession no. KU301747). The order and gene organization of *E. suratensis* mitogenome is in accordance with other vertebrates (Broughton & Dowling 2001; Wang et al. 2007). *ND6* gene and eight tRNA genes (*tRNA^Gln^*, *tRNA^Ala^*, *tRNA^Asn^*, *tRNA^Cys^*, *tRNA^Tyr^*, *tRNA^Ser^*, *tRNA^Glu^* and *tRNA^Pro^*) are encoded on the L-strand and remaining genes are encoded on the H-strand like *Heteropneustes fossilis* (Sahoo et al. 2016), *Labeo rohita*, *Catla catla* and *Cirrhinus mrigala* (Bej et al. 2012a,b; Bej et al. 2013). The overall base composition of the *E. suratensis* mitogenome on the heavy strand is as follows: A: 28.3%, C: 30.9%, G: 15.2%, T: 25.6% and A + T content: 53.9%. Frequently ATG is used as the start codon in all the protein-coding genes except *COI* which uses GTG. Stop codons used by the protein-coding genes are: TAA (*ND1*, *COI*, *ATP8*, *ND4L* and *ND5*), TAG (*ND6*) and incomplete stop codons (*ND2, COII, ATP6, CO3*, *ND4, ND3* and *Cytb*). In other teleost species, the mechanism of using an incomplete stop codon for stopping protein translations has been observed (Peng et al. 2006). The size of the tRNA genes ranged from 67 to 75 bp. Overlapping of nucleotides were observed in between *tRNA^Gln^* and *tRNA^Met^* (1 bp), ATP8 and ATP6 (10 bp), *ND4L* and *ND4* (7 bp), *ND5* and *ND6* (4 bp). Six gaps (1 bp to 37 bp) are observed in *tRNAs*. The two ribosomal RNA genes of *E. suratensis, 12S rRNA* (948 bp) and *16S rRNA* (1691 bp) are located between *tRNA^Phe^* and *tRNA^Leu^* and separated by *tRNA^Val^* like *Labeo fimbriatus* (Sahoo et al. 2015). Similarly the *control region* is located between *tRNA^Pro^* and *tRNA^Phe^*and observed to be 789 bp in length. As observed in other cyprinids the control region of *E. suratensis* contains a putative terminal associated sequence, three conserved sequence blocks (CSBI, CSBII and CSBIII) and TATA box. To validate the phylogenetic position of E. suratensis, we used MEGA6 software (MEGA Inc., Englewood, NJ) to construct a Neighbour-joining tree (with 500 bootstrap replicates) containing complete mitogenomes of 10 species derived from 7 different genus in cichlidae and *L. rohita* (Cyprinidae) was used as an outgroup to contrast the tree topology. Result shows a high degree of similarity among the cichlidae family and the two *Etroplus* genus, i.e. Green chromide and Orange chromide were in one branch (Figure 1). The information obtained from the present study would augment investigation of the population genetics and phylogenetics of the Cichlidae family.
